# Leaf meristems: an easily ignored component of the response to human disturbance in alpine grasslands

**DOI:** 10.1002/ece3.2059

**Published:** 2016-03-04

**Authors:** Jiangtao Hong, Xingxing Ma, Xiaodan Wang

**Affiliations:** ^1^ Institute of Mountain Hazards and EnvironmentChinese Academy of SciencesChengdu610041China; ^2^ University of Chinese Academy of SciencesBeijing100049China

**Keywords:** Alpine plant, biomass allocation, fencing, grazing, leaf meristems, Tibetan Plateau

## Abstract

Grazing and fencing are two important factors that influence productivity and biomass allocation in alpine grasslands. The relationship between root (R) and shoot (S) biomass and the root:shoot ratio (R/S) are critical parameters for estimating the terrestrial carbon stocks and biomass allocation mechanism responses to human activities. Previous studies have often used the belowground:aboveground biomass ratio (*M*_b_/*M*_a_) to replace the R/S in alpine ecosystems. However, these studies may have neglected the leaf meristem biomass, which belongs to the shoot but occurs below the soil surface, leading to a significant overestimation of the R/S ratio. We conducted a comparative study to explore the differences between the R/S and *M*_b_/*M*_a_ at both the species (*Stipa purpurea*,* Carex moorcroftii,* and *Artemisia nanschanica*) and community levels on a Tibetan alpine grassland with grazing and fencing management blocks. The results revealed that the use of the *M*_b_/*M*_a_ to express the R/S appeared to overestimate the actual value of the R/S, both at species and community levels. For *S. purpurea*, the *M*_b_/*M*_a_ was three times higher than the R/S. The *M*_b_/*M*_a_ was approximately two times higher than the R/S for the species of *C. moorcroftii* and *A. nanschanica* and at the community level. The relationships between the R‐S and *M*_b_‐*M*_a_ exhibited different slopes for the alpine plants across all the management practices. Compared to the fenced grasslands, the plants in the grazing blocks not only allocated more biomass to the roots but also to the leaf meristems. The present study highlights the contribution of leaf meristems to the accurate assessment of shoot and belowground biomasses. The R/S and *M*_b_/*M*_a_ should be cautiously used in combination in the future research. The understanding of the distinction between the R‐S and *M*_b_‐*M*_a_ may help to improve the biomass allocation mechanism response to human disturbances in an alpine area.

## Introduction

Human disturbances (i.e., grazing and fencing) have vital effects on the dynamics of grassland ecosystems that include the maintenance of species diversity, community structure and productivity (Grimes 1979; Tilman 1988; Hobbs and Huenneke 1992; Wu et al. 2009). The relationship between root (R) and shoot (S) biomass and the root:shoot ratio (R/S) are critical parameters for estimating the terrestrial carbon stocks and biomass allocation mechanism responses to human activities (Sun et al. 2014; Xiong et al. 2014). For example, previous studies have found significantly increased root proportions and R/S values due to grazing pressure (Van der Maarel and Titlyanova 1989; Li et al. 2008; Sun et al. 2014). Nonetheless, most of those studies conducted field sampling based on the ground surface, used the aboveground biomass:belowground biomass ratio (*M*_b_/*M*_a_) to replace the R/S and possibly misinterpreted the two ratios (Li et al. 2008; Evju et al. 2009; Yang et al. 2009, 2010; Wei et al. 2011; Sun et al. 2014; Xiong et al. 2014; Zhu et al. 2015). In most alpine graminoids and herbaceous plants, portions of the shoots (i.e., the leaf meristems or premature leaves, hereafter called leaf meristems in accordance with Körner (2003)) are located several centimeters below the soil surface to protect them from deleterious freezing temperatures because of the smaller variation in temperature of the soil than that of the atmosphere (Körner 2003). Furthermore, leaf meristems also could protect the vegetation against large herbivorous (Körner 2003).

Leaf meristems are very common for herbaceous plant including nearly all grasses and sedges in alpine ecosystems (from Körner (2003) and our observation). For example, Körner (2003) found that half of the shoot biomass (e.g., *Carex curvula* and *Ranunculus glacialis*) was located below the earth surface for alpine plant in the Andes Mountains, South America. In most sampling process, collecting plant productivity base on the ground is simply for convenience. However, this universal sampling method assumed that root and shoot are identified by the soil surface. It is likely to result in imprecise estimates on root and shoot biomass when considering the part of leaf meristems.

Fencing and grazing are two main managements on the alpine grasslands. Accurate assessment of the R/S is very important for estimating plant adaptations and survival strategies to the intensity of human activities. Increasing proportion of shoot biomass (decreasing R/S) allocated in fencing grasslands may indicate a stronger competition for light, while higher proportion of biomass allocated in root (low value of R/S) would suggest an intensified competition for soil nutrients (Lipowsky et al. 2011; Kiær et al. 2013). Thus far, however, knowledge of the belowground biomass remains limited due to the methodological difficulties associated with sampling and measuring root biomass (Vogt et al. 1996; Titlyanova et al. 1999). Furthermore, few studies have reported on important components of plant biomass, such as leaf meristems, which are closely linked to both the shoot and belowground biomass responses to different management practices because these components are easily ignored in alpine areas. The lack of leaf meristems studies may have resulted in the overestimation of the root biomass, root nutrient pools and R/S, which constitutes a large gap in our understanding of biomass restoration and allocation in alpine grasslands.

The aims of this study were to investigate the differences between the R/S and *M*_b_/*M*_a_ at both the species and community levels when the leaf meristems are taken into account. Next, we clarified the effects of grazing and fencing on leaf meristem biomass allocation in a case study conducted on the Tibetan Plateau.

## Methods

### Study sites

The Tibetan Plateau is the highest plateau in the world, and >60% of the region is covered by alpine grassland (Li and Zhou 1998). This alpine grassland is typical of the alpine ecosystems that play important environment roles throughout Asia and is extremely sensitive to human disturbances and climate change (Harris 2010). The study region was located in Shantsa County on the northern Tibet Plateau (30°56′ N, 88°41′ E, 4650 m a.s.l.). This area is a typic alpine grassland in the Tibet Autonomous Region of China. The mean annual temperature of the study area is 0°C, and the mean annual precipitation is 300 mm. *Stipa purpurea* and *Carex moorcroftii* are the dominant species, and the accompanying species include *Artemisia nanschanica*,* Leontopodium alpinum*, and *Oxytropis glacialis*. The area was used for yak and sheep grazing before the experimental blocks were established. Typically, this alpine steppe was a sparse grassland with low vegetation coverage (below 30%).

### Experimental design

The following four management blocks were set in this study: the grazing experimental pasture was used for typical grazing; the F1 experimental pasture was fenced for 1 year (since 2013); the F3 experimental pasture was fenced for 3 years (since 2011); and the F4 experimental pasture was fenced for 4 years (since 2010). All four blocks (grazing, F1, F3 and F4) were on flat terrain and located within 1000 m of each other. Each fencing block was enclosed with barbed wire to prevent livestock farming and covered an area of approximately 1000 m^2^. Then, four subplots (10 m × 10 m) were set in each fencing and grazing blocks. The grazing intensity of the grazing block was approximately 120 sheep units per km^2^ (one Tibetan sheep is equivalent to one sheep unit, while one yak is approximately equivalent to five sheep units based on their feed intakes; Yan et al. 2005).

### Field sampling

The samples were collected in August of 2014 when the pastures were at annual peak biomass. Individuals of *S. purpurea, C. moorcroftii* and *A. nanschanica* were randomly selected using the line transect method from each of the management plots. These three species were accounted for a large proportion of the biomass of the community. Soil cores (15 cm in width, 15 cm in length and 30–50 cm in depth) that contained only a single targeted individual were removed with a shovel while ensuring the integrities of the plants (twenty individuals for the three species in grazing, F1, F3 and F4, respectively). In total, 240 individual whole plants were sampled. Furthermore, four quadrats (1 m × 1 m) were randomly selected in four subplots in each management blocks for the collection of the community biomasses. For each of these 16 quadrats, all of the community biomass (roots and shoots) was harvested via digging with a shovel to a depth that matched that of the visible roots (30–50 cm).

### Biomass measure

**Figure 1:**
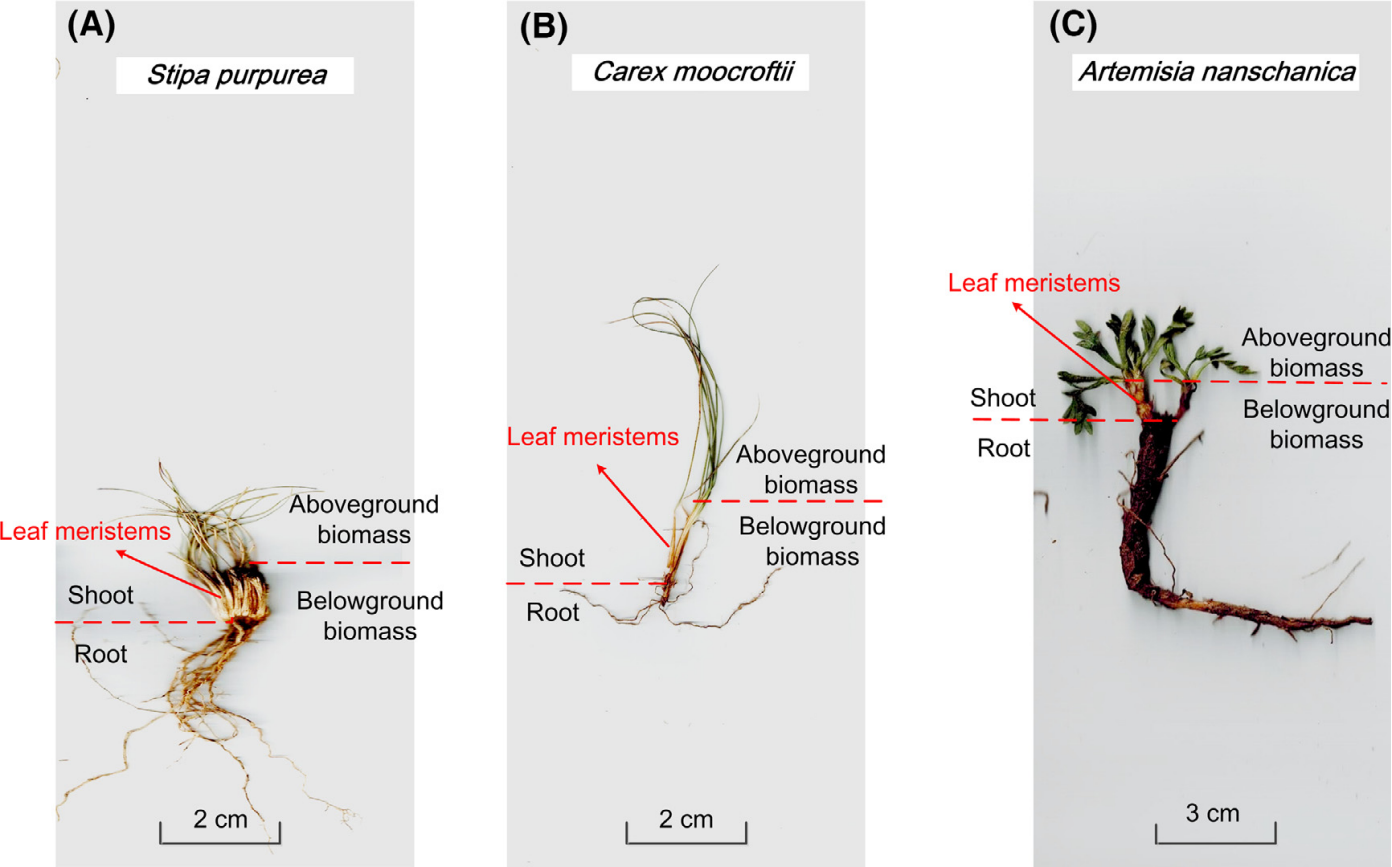
Components of the plant biomasses of *Stipa purpurea*,*Carex moorcroftii,* and *Artemisia nanschanica* on a Tibetan alpine grassland.

All of the individual and community biomass samples were immediately taken to the laboratory and carefully washed. The *S. purpurea*,* C. moorcroftii*, and *A. nanschanica* individuals and community biomass were separated into three parts, including aboveground biomass, leaf meristems and root (Fig. 1). The aboveground biomass and leaf meristems were easily identifiable by color. Because the leaf meristems were situated underground throughout year, they were usually white rather than green (Körner 2003). Morphology and color were the important characteristics to distinguish leaf meristems from root. The individual and community biomasses were oven‐dried at 65°C to a constant weight. The aboveground, leaf meristem and root biomasses were directly weighed with an electronic balance with an accuracy of 0.001 g. The belowground biomass was calculated by adding the root and leaf meristem biomasses, and the shoot biomass was calculated by adding the aboveground and leaf meristem biomasses (Fig. 1). The belowground and shoot biomass fraction (%) was also calculated by the same method. It is worth to note that the community biomass was collected base on the quadrat (g m^−2^), while the three species (*S. purpurea*,* C. moorcroftii*, and *A. nanschanica*) were collected base on one individual plant (g).

### Statistical analyses

The differences between the R/S and *M*_b_/*M*_a_ for *S. purpurea*,* C. moorcroftii*,* A. nanschanica* and the community samples were analyzed with paried *t*‐tests. The relationships of the root‐shoot and belowground‐aboveground biomasses were examined with simple linear regression analyses. The differences between the aboveground, leaf meristem and root biomasses in the four management blocks for *S. purpurea*,* C. moorcroftii*,* A. nanschanica* and the community samples were analyzed with one‐way ANOVA. Where necessary, the data were log transformed to meet normal distribution and heteroscedasticity requirements. The statistical analyses were performed using SPSS, ver. 16.0 (SPSS Inc., Chicago, IL), and the figures were plotted using the SigmaPlot 11.0 software (Systat Software, Inc., Richmond, CA).

## Results and Discussion

### Difference between *M*_b_/*M*_a_ and R/S in the alpine grassland

**Figure 2:**
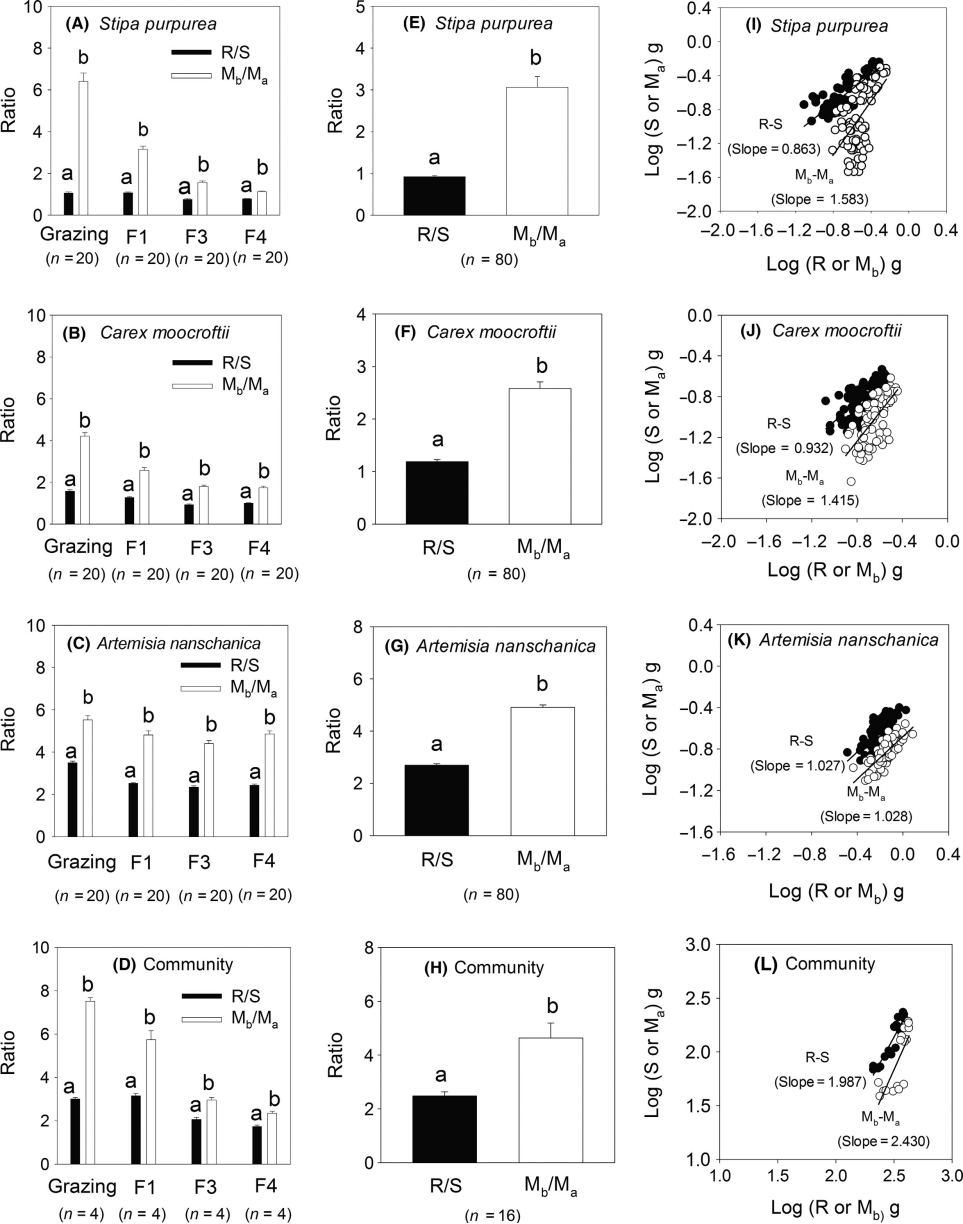
Differences between the R/S and *M*_b_/*M*_a_ at both the species and community level for each of four management blocks (A–D). The differences between R/S and *M*_b_/*M*_a_ for each same species and the combined community (E–H). The relationships of the belowground‐aboveground biomasses and root‐shoot biomasses at both the species and community levels on the Tibetan alpine grassland (I–L). All regression lines are shown for the relationships that were significant at *P *<* *0.0001. Different letters indicate significant differences between the R/S and *M*_b_/*M*_a_ values (*P *<* *0.05).

The *M*_b_/*M*_a_ was significantly higher than the R/S at both the species and community levels across the four management blocks (*P *<* *0.05; Fig. 2A–D). When the individuals of the same species (*S. purpurea*,* C. moorcroftii*, and *A. nanschanica*) and the 16 quadrats of community were aggregated, the *M*_b_/*M*_a_ was also higher than the R/S (*P *<* *0.05; Fig. 2E–H). The use of *M*_b_/*M*_a_ to express R/S appeared to overestimate the actual value of the R/S in the alpine grassland at both the species and community levels. For *S. purpurea*, the *M*_b_/*M*_a_ was three times higher than the R/S. The *M*_b_/*M*_a_ was approximately two times higher than the R/S for species of *C. moorcroftii* and *A. nanschanica* and at the community level. Many studies have ignored the proportion of the leaf meristem biomass, which belongs to the shoot portion but is located below the soil surface. In the alpine ecosystems, the leaf meristems are buried several centimeters below the soil surface. In addition, the buried depth differed in plant species and phylogeny. The leaf meristems of grasses and sedges (e.g., *S. purpurea* and *C. moorcroftii*) are buried 1–3 cm below the ground, while leaf meristems of some cushion plant (e.g., *Arenaria pulvinata*) are only at a depth of 0.5–1 cm. The diurnal variations in soil temperature are small, and thus, buried meristems are typical evidence of the morphological avoidance of extremely low temperatures (Körner 2003). Furthermore, this allocation mechanism is also conducive to avoiding the zone of severe surface heating during times of intense direct solar radiation, large grazing animals and mechanical damage (Körner 2003). The allocation is a vital survival mechanism for plants to overcome extreme alpine environments.

### Difference between *M*_b_‐*M*_a_ and R‐S relationships in the alpine grassland

The R‐S and *M*_b_‐*M*_a_ relationships exhibited different slopes for the *S. purpurea*,* C. moorcroftii* and community samples (Fig. 2I, J, L). The slope between the root and shoot biomass is a key parameter for exploring biomass allocation mechanisms (Cheng and Niklas 2007; Yang et al. 2009). If we had not accounted for the leaf meristems, the *M*_b_‐*M*_a_ would have been the same as the R‐S, which would have been consistent with previous studies. However, leaf meristems are ubiquitous in alpine plants and should be considered to compose an important proportion of the biomass. The *M*_a_ and shoot values were different as the *M*_b_ and root values. The distinctions between the *M*_b_ and root and between the *M*_a_ and shoot may be important factors that could influence the reliability of the results (Mokany et al. 2006; Yang et al. 2010). For example, Yang et al. (2009) found that an isometric hypothesis could be applied to describe alpine grass biomass distributions based on the *M*_b_ and *M*_a_ values. In fact, the slopes of the R‐S and *M*_b_‐*M*_a_ relationships were different in alpine ecosystems, and thus, the conclusions of these authors are questionable. The two relationships (R‐S and *M*_b_‐*M*_a_) should be cautiously combined when examining alpine plants. The understanding of the distinction between the R‐S and *M*_b_‐*M*_a_ relationships may reflect improvements in biomass allocation mechanisms in alpine areas. If the belowground biomass was identified with root biomass, it would result in an overestimation in nutrients pools of the root. On the contrary, the shoot nutrient pools would be underestimated because some shoot tissues (e.g., leaf meristems) were buried in the ground. In this study, we have not investigated the nutrient concentrations of the leaf meristems, while the nutritional characteristic of this important plant component will be considered in our future research.

### Effect of grazing and fencing on biomass allocation in the alpine grassland

**Figure 3:**
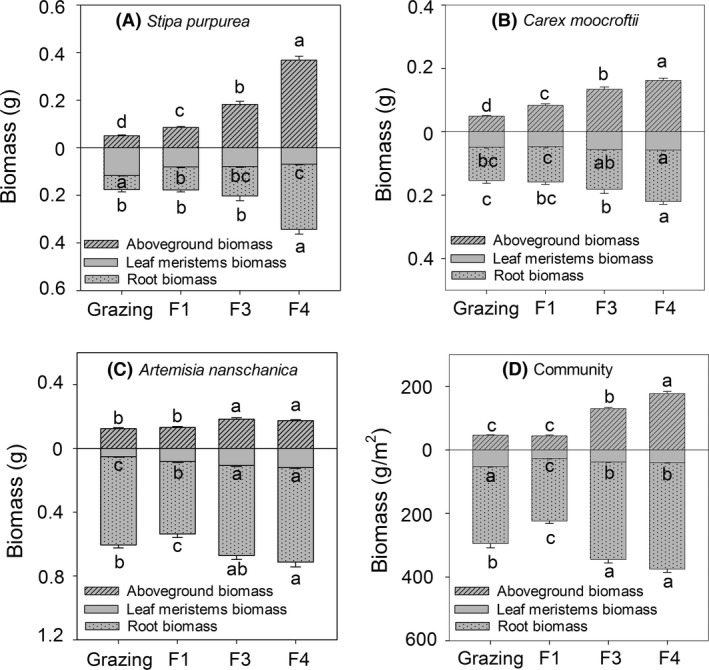
Biomass allocations (aboveground, leaf meristems, and root biomasses) of *Stipa purpurea*,*Carex moorcroftii*,*Artemisia nanschanica* and the combined community. The different letters indicate biomasses were significantly different between the four blocks (*P *<* *0.05).

**Figure 4:**
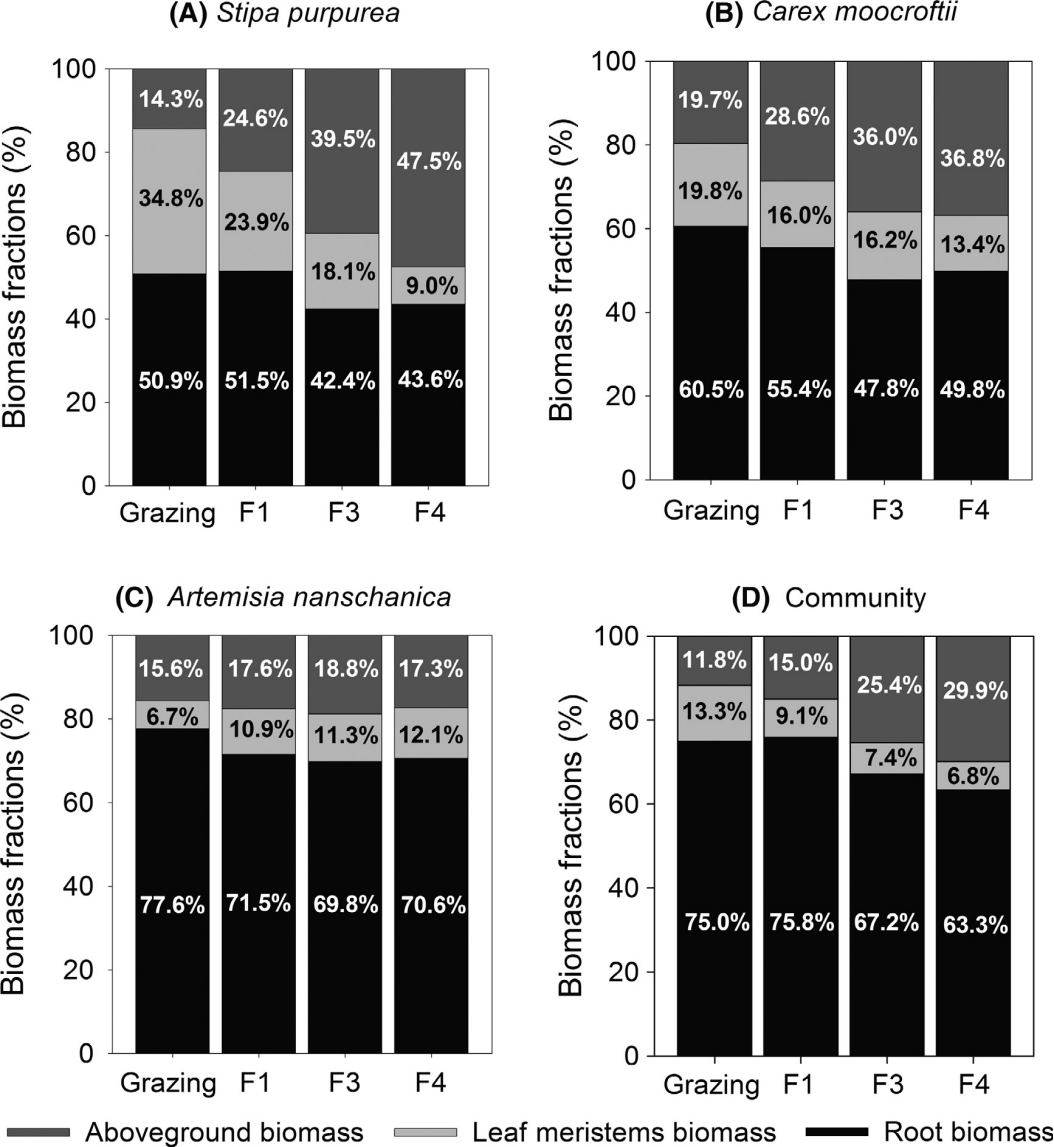
Biomass fractions (aboveground, leaf meristems, and root biomasses) of *Stipa purpurea*,*Carex moorcroftii*,*Artemisia nanschanica* and the combined community. The different letters indicate biomasses that were significantly different between the four blocks (*P *<* *0.05)

The grazing block consistently exhibited lower aboveground and root biomasses than the blocks that had been fenced for long durations (F3 and F4) at the species and community levels (*P *<* *0.05; Fig. 3A–D). However, the leaf meristem biomasses of the individual species exhibited different responses to labor management. The *S. purpurea* in the grazing and short‐term fenced blocks (i.e., the grazing and F1 blocks) exhibited greater leaf meristem biomasses than those in the long‐term fenced blocks (i.e., F3 and F4). However, the *C. moorcroftii* and *A. nanschanica* samples from the grazing block exhibited lower leaf meristem biomasses than the samples from F3 and F4 (Fig. 3B, C). Compared to the grazing block, the fenced grasslands exhibited reduced leaf meristem biomasses at the community level (Fig. 3D). The aboveground biomass fractions of the *S. purpurea*,* C. moorcroftii,* and community samples were increased, but the leaf meristem biomass percentages were decreased after 1 year of enclosure with a fence (Fig. 4A, B, D). For *A. nanschanica*, the fractions of leaf meristem biomass in the fenced blocks were higher than that in the grazing block (Fig. 4C). The F3 and F4 blocks always exhibited lower fractions of belowground biomass than the grazing and F1 blocks (Fig. 4A, B, D), with the exception of the *A. nanschanica* samples (Fig. 4C).

Both the amount and fraction of aboveground biomass were increased after grazing exclusion, which is consistent with previous studies (Sun et al. 2014; Xiong et al. 2014). On one hand, fewer photosynthetic organs were eaten by livestock following the removal of grazing. On the other hand, the increase in the biomass allocated above ground may have been due to increased competition for light due to the development of vegetation coverage (increasing from approx. 12% in grazing blocks to approx. 30% in F4 blocks) (Lipowsky et al. 2011; Kiær et al. 2013). The amount and fraction of the leaf meristem biomass of the *S. purpurea* biomass were decreased by enclosed management. For the *S. purpurea* and *C. moorcroftii* species and community biomasses, the leaf meristem proportions accounted for even higher levels of biomass than the aboveground portion in the grazing block (Fig. 4A, B, D). Thus, the most striking divergence between the *M*_b_/*M*_a_ and R/S values was observed in grazing grasslands. In this study, *S. purpurea* was the most important palatable forage, and thus, the individuals of this species allocated more biomass below the soil surface as protection mechanism against a high grazing pressure to ensure long lives (Körner 2003). Furthermore, the present results suggest new perspective that grazing may result not only in the allocation of more biomass to roots but also to the leaf meristems in alpine ecosystem. Nevertheless, a similar phenomenon was not observed for the unpalatable herbage of *A. nanschanica*. In our case, significantly greater *M*_b_/*M*_a_ values were observed in the grazing plots, which is consistent with the results of a study conducted in the Swedish steppe in which an increased allocation of resources to the belowground biomass was observed in overgrazing conditions (Van der Maarel and Titlyanova 1989).

## Conclusion

To the best of our knowledge, this is the first study to explore the leaf meristem responses to grazing and fencing. The present study has provided some useful information that distinguishes the *M*_b_/*M*_a_ and R/S ratios in alpine areas. More attention should be given to the role of leaf meristem biomass, which is an important component of the shoots but is located below ground. The *M*_b_/*M*_a_ ratio, as calculated with the general sampling method based on the soil surface, cannot replace the R/S ratio in alpine areas. A reduced fraction of belowground biomass was observed after grazing exclusion due to decreased root and the leaf meristem proportions. Our results highlight the contribution of leaf meristems to evaluations of shoot and belowground biomasses in alpine grasslands.

## Conflict of Interest

None declared.
